# Transcriptomic Analysis of Subtype-Specific Tyrosine Kinases as Triple Negative Breast Cancer Biomarkers

**DOI:** 10.3390/cancers15020403

**Published:** 2023-01-07

**Authors:** Praopim Limsakul, Pongsakorn Choochuen, Gorn Charupanit, Krit Charupanit

**Affiliations:** 1 Division of Physical Science, Faculty of Science, Prince of Songkla University, Songkhla 90110, Thailand; 2 Center of Excellence for Trace Analysis and Biosensor (TAB-CoE), Prince of Songkla University, Songkhla 90110, Thailand; 3 Department of Biomedical Sciences and Biomedical Engineering, Faculty of Medicine, Prince of Songkla University, Songkhla 90110, Thailand; 4 Department of Pathology, Faculty of Medicine, Chulalongkorn University, Bangkok 10330, Thailand; 5 Department of Pathology, Pattani Hospital, Pattani 94000, Thailand

**Keywords:** triple negative breast cancer, tyrosine kinase, cancer marker identification

## Abstract

**Simple Summary:**

Triple negative breast cancer (TNBC) is the most aggressive subtype of breast cancer, with a higher mortality rate when compared to the other subtypes. Because of the absence of a molecular target and the heterogeneity of TNBC molecular subtypes, the development of targeted therapies is limited. Protein tyrosine kinases (TKs), in particular, have emerged as important molecular targets and biomarkers in cancer. Thus, we identified the signature TK of individual TNBC subtypes by analyzing RNA-seq-based transcriptome data of TNBC patients from The Cancer Genome Atlas database and validated this finding against other TNBC patients and cell line datasets. Our discovery of the signature TK for TNBC subtypes can be used as actionable targets for anti-cancer therapies, as well as subtype-specific biomarkers for TNBC.

**Abstract:**

Triple negative breast cancer (TNBC) shows impediment to the development of targeted therapies due to the absence of specific molecular targets. The high heterogeneity across TNBC subtypes, which can be classified to be at least four subtypes, including two basal-like (BL1, BL2), a mesenchymal (M), and a luminal androgen receptor (LAR) subtype, limits the response to cancer therapies. Despite many attempts to identify TNBC biomarkers, there are currently no effective targeted therapies against this malignancy. In this study, thus, we identified the potential tyrosine kinase (TK) genes that are uniquely expressed in each TNBC subtype, since TKs have been typically used as drug targets. Differentially expressed TK genes were analyzed from The Cancer Genome Atlas (TCGA) database and were confirmed with the other datasets of both TNBC patients and cell lines. The results revealed that each TNBC subtype expressed distinct TK genes that were specific to the TNBC subtype. The identified subtype-specific TK genes of BL1, BL2, M, and LAR are *LYN*, *CSF1R*, *FGRF2*, and *SRMS*, respectively. These findings could serve as a potential biomarker of specific TNBC subtypes, which could lead to an effective treatment for TNBC patients.

## 1. Introduction

Breast cancer is a heterogeneous group of diseases. Expression of hormone receptors, such as estrogen receptor (ER) and progesterone receptor (PR), or human epidermal growth factor receptor 2 (HER2), can indicate the probability of responsiveness to therapies targeting these receptor proteins. Triple negative breast cancer (TNBC), which is approximately 15–20% of all breast cancer cases, shows an impediment to the development of targeted therapies due to the absence of molecular targets [1,2]. TNBC displays transcriptional diversity with four tumor-intrinsic subtypes, including two basal-like (BL1, BL2), a mesenchymal (M), and a luminal androgen receptor (LAR) subtype [3]. Each subtype displays unique biology that affects treatment efficacy in different ways; for example, only a subset of TNBC patients respond to the standard-of-care chemotherapy [4,5,6] or the immune checkpoint blockade treatment, which is one of the most effective current treatments for TNBC patients [7,8]. Because of the heterogeneity of TNBC, it is challenging to understand the underlying disease mechanisms and design targeted treatments. Further analysis of the molecular basis for differences among TNBC subtypes will help to uncover actionable targets to improve the treatment outcome.

A potential target that is generally used for the prognostic marker, as well as cancer therapy, is a protein kinase because of its association with human cancer initiation and progression [9,10]. Among over 500 protein kinases, the human genome contains approximately 90 protein tyrosine kinases (TKs), which can regulate cellular proliferation, survival, differentiation, function, and mobility [10]. TKs can be classified into the receptor tyrosine kinase (RTK), which consists of transmembrane proteins with a ligand-binding extracellular domain and a catalytic intracellular kinase domain, and the non-receptor tyrosine kinase (NRTK), which is found in the cytosol. Examinations of genomic, transcriptomic, and proteomic profiling, as well as immunohistochemistry of TNBC cohort and cell line samples, revealed that TKs could be potential prognostic and/or therapeutic targets for TNBC. The majority of reported TKs that are highly expressed in TNBC are RTKs, such as the ErbB family (e.g., EGFR, ERBB4) [11,12,13,14], C-Kit [15,16], PTK7 [17], VEGFR2 [18], PDGFRα [18], and the EPH-receptor family (e.g., EPHA1, EPHA4, EPHA7, EPHB4, and EPHB6), FLT1, ALK, and PTK2B [19]. In addition to RTKs, the cytoplasmic TKs belonging to the SRC family kinases (e.g., SRC and FYN) and immune-related genes (e.g., ITK and ZAP70) were found to be highly expressed in TNBC [20].

Over the past three decades, the FDA has approved at least 24 new therapeutic drugs for breast cancer, including 18 small molecules, three monoclonal antibodies (mAbs), and three antibody-drug conjugates (ADCs) [21,22,23]. Each one is specific to stages and subtypes of breast cancer. The majority of them are prescribed to patients diagnosed with hormone receptor-positive breast cancers (i.e., ER- and/or PR-positive breast cancers) and HER2-positive breast cancer. Only three drugs received accelerated approval for the first therapies of metastatic TNBC, including two mAbs and an ADC [24]. Atezolizumab (in combination with paclitaxel) and pembrolizumab (in combination with chemotherapy) are mAbs that target the programmed cell death protein 1 (PD-1), whereas sacituzumab govitecan-hziy is an ADC that combines an antibody targeting the human trophoblast cell-surface antigen 2 (Trop-2) and a cytotoxic drug, SN-38. In addition, targeting protein kinases with small molecules or antibodies for the treatment of various types of cancer, including breast cancer, has been successfully proven in clinical trials. EGFR is typically used as a potential therapeutic target for TNBC since it is frequently overexpressed in TNBC compared to other breast cancer subtypes [25]. However, the clinical trials of EGFR inhibitors, including small molecules and mAbs, have not been satisfactory due to low response rates from the genetic heterogeneity of TNBC subtypes [25,26]. Inhibiting multiple kinases against a variety of growth factor receptors, including platelet-derived growth factor receptor (PDGFR), fibroblast growth factor receptors (FGFRs), vascular endothelial growth factor receptor (VEGFR), as well as the proto-oncogenes RET, FLT3, and SRC, with anti-angiogenic activity, nintedanib has shown efficacy in early clinical trials for TNBC [27].

Although TKs are commonly activated in cancers and serve as important targets for anti-cancer therapies, as well as predictive markers for therapeutic response [9], there are currently no effective therapies associated with TKs for TNBC. Given the distinct genomic alterations and clinicopathological characteristics of different TNBC subtypes, investigation of subtype-specific TK changes could reveal molecular vulnerabilities within TNBC that potentially lead to novel therapeutic targets. Differential expression analysis of kinase genes expressing in TNBC has been studied using RT-PCR, microarrays, or RNA-seq [3]; however, the unique TKs in each TNBC subtype have not been reported. In this study, the RNA-seq data from TCGA of TNBC cohorts [28] were analyzed to illustrate the transcriptome profile in TNBC, with a focus on TK genes. We discovered TK genes that are differentially expressed (DE) in different TNBC subtypes. The gene and kinase enrichment analyses, including Gene Ontology (GO) and Kyoto Encyclopedia of Genes and Genomes (KEGG) pathway enrichment analyses, were performed to reveal distinct biological mechanisms and functions of each subtype, and Kinase Enrichment Analysis 3 (KEA3) was used to further predict the upstream kinases based on the DE genes. Our findings were validated by other RNA expression datasets from TNBC patients and cell lines. Overall, this study provides in-depth transcriptomic-based TK profiles of TNBC and identifies subclass-specific TKs of each TNBC subtype that could lead to the improvement of antitumor efficacy, as well as novel TK targets for clinical exploration and therapeutic application.

## 2. Materials and Methods

### 2.1. RNA-Seq Datasets

We performed analysis on publicly available transcriptomic data from TCGA [28] (UCSC XENA Project—https://xenabrowser.net/, accessed on 17 July 2022). The transcripts per million (TPM) RNA-seq data of TNBC patients were selected based on the TCGA-BRCA project [28] and further classified into four subtypes [3]. The classification of the subtype was performed using the TNBCtype web-based tool (http://cbc.mc.vanderbilt.edu/tnbc/, accessed on 17 July 2022), based on the highest positive centroid correlation and *p*-value on normalized mRNA expression of TNBC samples. In total, 183 TNBC samples were assigned to either BL1 (64 samples), BL2 (37 samples), M (54 samples), or LAR (28 samples) subtypes. 

### 2.2. Gene Expression Analysis

Differential gene expression analysis of RNA-seq data, excluding long noncoding RNAs (lncRNAs), was performed using R package DESeq2 (version 1.30.1) [29]. To identify subtype-specific genes in each TNBC subtype, we performed differential gene expression analysis using a subtype of interest versus the remaining three TNBC subtypes (subtype/TNBC). Identified genes with adjusted *p*-value < 0.05 were considered statistically significant and further used to quantify their expression based on log_2_ fold change (log2FC). The criteria to select the DE gene specific to each TNBC subtype was set as |log2FC| ≥ 1, where upregulated and downregulated genes were defined as the genes with log2FC ≥ 1 and log2FC ≤ −1, respectively. We also employed Venn, the online tool (http://bioinformatics.psb.ugent.be/webtools/Venn/, accessed on 30 July 2022), to generate a Venn diagram to show the relation of DE genes among TNBC subtypes.

### 2.3. Gene Ontology and KEGG Pathway Enrichment Analysis

To further investigate DE genes in each TNBC subtype, GO enrichment analysis and KEGG pathway analyses were performed. Upregulated DE genes from each TNBC subtype (*p*-value < 0.05 and log2FC ≥ 1) were analyzed using the functional annotation tool, that is, the Database for Annotation, Visualization, and Integrated Discovery (DAVID, https://david.ncifcrf.gov/, accessed on 30 July 2022) [30]. The default parameters with medium stringency were implemented and computed over the background of the whole human genome. The results from GO and KEGG with Fisher’s exact *p*-values < 0.05 were selected for further analysis.

### 2.4. Kinase Enrichment Analysis

Upregulated DE genes in each TNBC subtype were converted into protein. Then, these proteins were used as the input to Kinase Enrichment Analysis 3, KEA3 (https://maayanlab.cloud/kea3, accessed on 13 August 2022), which is a web server application that infers overrepresentation of upstream kinases of which putative substrates are in a user-inputted list of proteins [31]. The kinases were ranked using the MeanRank score, and the top 20 kinases were reported. These kinases were then grouped into kinase families using KinMap, which is a web-based tool (http://www.kinhub.org/kinmap/, accessed on 13 August 2022) [32].

### 2.5. Gene Expression Validation

Other publicly available transcriptomic data of TNBC patients and cell lines from the following datasets were analyzed to validate the TCGA results. The Clinical Proteomic Tumor Analysis Consortium (CPTAC) dataset [33] includes mass spectrometry-based proteomics with next-generation DNA and RNA sequencing profiles from 122 treatment-naive primary breast cancer patients, in which 27 samples were classified as BL1 (11 samples), BL2 (3 samples), M (9 samples), and LAR (4 samples) TNBC subtypes [3]. The MET500 dataset [34] contains gene expression profiles of 500 cancer patients with metastatic cancers from more than 30 primary sites and biopsied from over 22 organs, in which 39 breast cancer samples were classified as BL1 (11 samples), BL2 (10 samples), M (8 samples), and LAR (10 samples) TNBC subtypes [3]. The Molecular Taxonomy of Breast Cancer International Consortium (METABRIC) dataset [35] consists of normalized RNA microarray profiling on 1981 fresh-frozen primary breast cancer specimens, in which 325 samples were specified as BL1 (119 samples), BL2 (63 samples), M (65 samples), and LAR (78 samples) TNBC subtype [3]. The Cancer Cell Line Encyclopedia dataset (CCLE, from DepMap Public 22Q2) [36] consists of gene expression profiles of 1406 cell lines, 83 of which are breast cancer cell lines. Only 20 TNBC cell lines were then selected for this study and classified as BL1 (5 samples, i.e., HCC1599, HCC1937, HCC38, HCC1143, and HCC2157), BL2 (5 samples, i.e., HCC1187, HCC1806, HCC70, CAL-85-1, and SUM149PT), M (5 samples, i.e., CAL-120, BT-549, MDA-MB-436, MDA-MB-157, and MDA-MB-231), and LAR (5 samples, i.e., BT-20, CAL-148, MDA-MB-453, SUM185PE, and MFM-223) TNBC subtypes. In total, we validated our findings with 391 TNBC patients and 20 TNBC cell lines, including 146 BL1, 81 BL2, 87 M, and 97 LAR samples.

To validate the results, the mRNA expression z-score of TK genes of interest in each dataset was used to compare the expression across all TNBC subtypes. Furthermore, the receiver operating characteristic (ROC) curve of those TK genes was used to illustrate the performance of identified TK genes in classifying the TNBC subtype from TNBC patient and cell line datasets. The ROC curve is made up of sensitivity (*y*-axis) and 1–specificity (*x*-axis). In addition, the area under the ROC curve (AUC), which ranges from zero to one (one indicating perfect performance), was used to represent the overall performance of identified TK genes as signature genes to classify TNBC subtypes.

### 2.6. Statistical Analysis

All data analyses were performed in R (version 4.0., R Foundation for Statistical Computing, Vienna, Austria). The statistical details of all experiments were reported within the text, in the figure legends, and in figures, including statistical analysis performed and statistical significance.

## 3. Results

### 3.1. Differentially Expressed Genes among TNBC Subtypes

**Figure 1:**
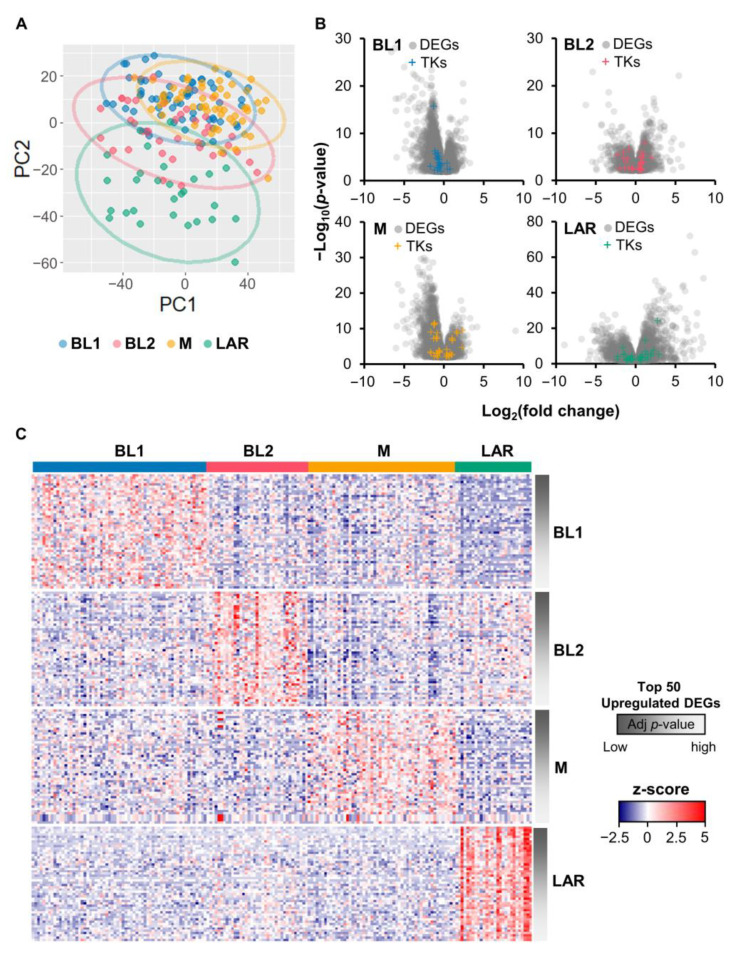
Differential gene expression analysis of TNBC samples from TCGA dataset. (**A**) Principal component analysis of mRNAs expression in each subtype, including BL1, BL2, M, and LAR. Each dot represented one TNBC cohort. (**B**) Volcano plot showing the gene expression differences between particular TNBC subtypes and the remaining subtypes. Gray dots represented genes with adjusted *p*-value < 0.05, whereas those with the plus sign (+) represented DE genes encoding protein tyrosine kinases. DEGs: differentially expressed genes. (**C**) Heatmap of the expression levels (z-score TPM) of top 50 upregulated DE genes in each subtype of TNBC. Rows were ascendingly ranked according to the adjusted *p*-value from the differential gene expression analysis (more to less significant), and columns represented individual cases. Red indicated overexpression, while blue indicated underexpression. (**A**–**C**) The BL1, BL2, M, and LAR subtypes were indicated in blue, pink, orange, and green, respectively.

The TCGA RNA-seq dataset of TNBC patients was analyzed with DESeq2 to identify DE genes [28,29]. Using genomic data guided by the expression distribution of clinically defied ER, PR, and HER2 tumors, about 17% of TCGA breast cancer patients were classified as TNBC. These TNBC samples were then further categorized into four subtypes, including BL1 (64 samples, 35%), BL2 (37 samples, 20%), M (54 samples, 30%), and LAR (28 samples, 15%) [3]. The results using principle component analysis showed the heterogenicity of TNBC subtypes on the transcriptome level (Figure 1A). Other principal components also illustrated the unclear differentiation of TNBC subtypes (Figure S1). However, LAR showed the most distinct separation from other subtypes (Figure 1A).

The DE genes of a particular TNBC subtype were defined when changes in gene expression levels of that particular subtype were higher than the remaining subtypes. Overall, 4217, 2602, 3902, and 5749 genes were differentially expressed with adjusted *p*-value < 0.05 in BL1, BL2, M, and LAR, respectively. Among these, there were 138 (BL1), 341 (BL2), 344 (M), and 966 (LAR) upregulated genes, and 970 (BL1), 341 (BL2), 1020 (M), and 1043 (LAR) downregulated genes (Figure 1B). Only upregulated DE genes were included for further analysis. The expression of the top 50 upregulated DE genes ranked based on the adjusted *p*-value in each TNBC subtype were illustrated in the heatmap (Figure 1C). Majority of TNBC patients showed similarity in RNA expression levels of those DE genes within the same subtype but difference across subtypes. This indicated the potential to identify the molecular signature of each TNBC subtype, regardless of the heterogenicity of TNBC patients.

### 3.2. Signature TK Genes Identification of Each TNBC Subtype

**Figure 2:**
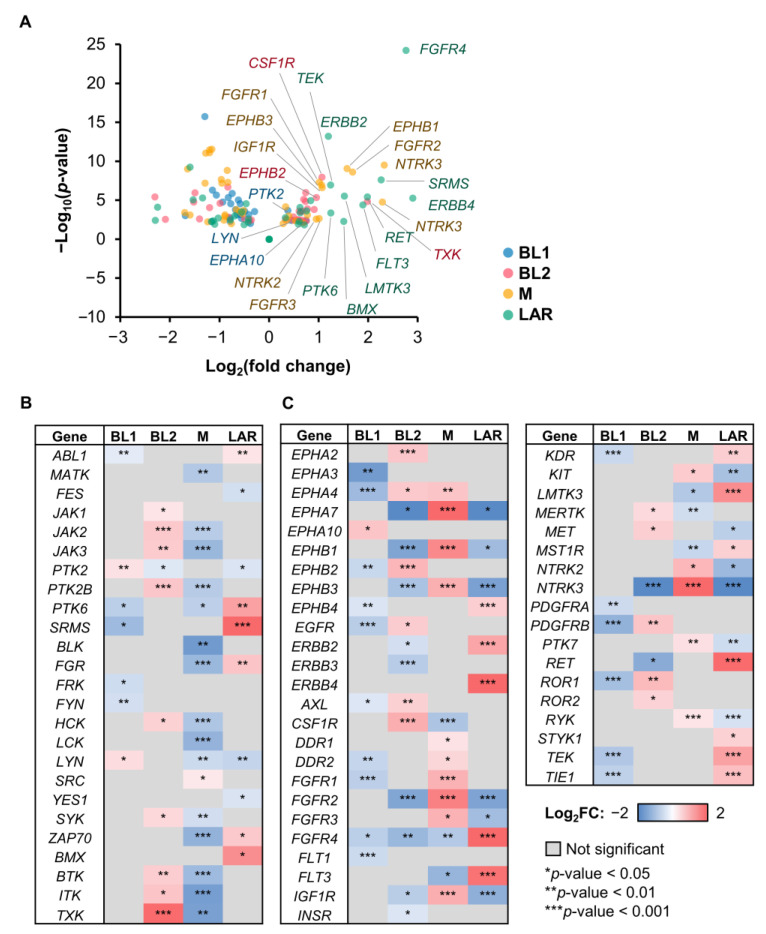
Differentially expressed TK genes showing signature TKs in each TNBC subtype. (**A**) Volcano plot showing the TK gene expression differences between a specific subtype and the rest of TNBC patient cohorts. Heatmap displaying the log2FC of the (**B**) NRTKs and (**C**) RTKs. Row represented DE TK genes with the adjusted *p*-value: * < 0.05, ** < 0.01, *** < 0.001, and column represented the TNBC subtype. The color bar from blue to red illustrated the level of fold change from low to high, where non-significant DE TK genes (adjusted *p*-value > 0.05) were highlighted in gray.

The signature TK gene was identified from differentially expressed TK genes by comparing a certain subtype to the remaining TNBC subtypes. Only upregulated TK genes were considered as subtype-specific signature TK genes (Figure 2A).

The log2FC distribution of NRTK genes revealed differences among TNBC subtypes (Figure 2B). Eighteen DE TK genes with positive fold change were found in BL1 (2 TKs), BL2 (9 TKs), M (1 TK), and LAR (6 TKs). Among these, the expression of NRTKs with log2FC > 1 was only observed in BL2 and LAR. We found that *TXK* had the highest fold change among upregulated TK genes in BL2, followed by the kinases of the Tec family (i.e., *BTK* and *ITK*) and the Janus family (i.e., *JAK1*, *JAK2*, and *JAK3*). In LAR, the kinases of the PTK6 family, including *SRMS* and *PTK6* (breast tumor kinase, *BRK*), were found to be upregulated, followed by *BMX* from the Tec family. On the other hand, upregulated kinase genes of the SRC family, such as *LYN* and *SRC*, were detected in BL1 and M, respectively.

Similar to NRTK, changes in RTK gene expression levels in each TNBC subtype were also unique (Figure 2C). Thirty-eight DE TK genes with positive fold change were found in BL1 (1 TK), BL2 (11 TKs), M (15 TK), and LAR (12 TKs), with *EPHA4* overlapping between BL2 and M. In BL1, only *EPHA10*, which is a class of Eph receptor RTKs, was found to be upregulated. In BL2, the top-upregulated kinase gene with the highest FC was *CSF1R*. Several RTKs of Eph family dominantly involved in BL2 in both upregulation (*EPHA2, EPHA4, EPHB2*) and downregulation (*EPHA7, EPHB1*, and *EPHB3*). Furthermore, EGFR was found to be significant in BL2. A variety of RTKs from different families, such as the Eph, DDR, FGFR, and NTRK families, were positively or negatively involved in the M subtype. In addition, *EPHA7*, *EPHB1*, *EPHB3*, *FGFR1*, *FGFR2*, *FGFR3*, *IGF1R*, and *NTRK3* were found to be upregulated, especially *EPHA7* and *NTRK3,* which showed the highest FC in M but the lowest in the BL2 and LAR subtypes. In LAR, seven RTKs, e.g., *ERBB2*, *ERBB4*, *FGFR4*, *FLT3*, *LMTK3*, *RET*, and *TEK*, were upregulated where *ERBB4* and *FGFR4* had the highest FC compared to the rest of TNBC subtypes. We also found that the expression of *ERBB2* (or *HER2*), which is typically used to classify one of the major types of breast cancer, was found to be upregulated in LAR.

Combining TK gene expression results from both NRTKs and RTKs, the top three TKs with the most significant and the highest FC in each subtype were *EPHA10*, *LYN*, and *PTK2* for BL1; *CSF1R*, *EPHB2*, and *TXK* for BL2; *EPHA1*, *FGFR2*, and *NTRK3* for M; *ERBB4*, *FGFR4*, and *SRMS* for LAR. These identified TKs could potentially be subtype-specific signature TK genes of the particular TNBC subtype.

### 3.3. Kinase Enrichment Analysis of DE Genes

To infer the potential upstream kinases in accordance with the upregulated DE genes in each TNBC subtype, we performed kinase enrichment analysis using KEA3 [31]. Searching through kinase-substrate databases covering more than 500 unique protein kinases, the top 20 upstream kinases based on the MeanRank of KEA3 were selected. These enriched kinases were then mapped in the phylogenetic tree of the human kinome using KinMap to categorize kinases into eight typical groups (AGC, CAMK, CK1, CMGC, STE, TK, TKL, Other) and 13 atypical families among the human kinome [37]. The distribution of enriched kinases revealed that the majority of kinase groups found in all TNBC subtypes was TK which appeared at 35% in BL1, 70% in BL2, 85% in M, and 55% in LAR (Figure S2). 

**Figure 3:**
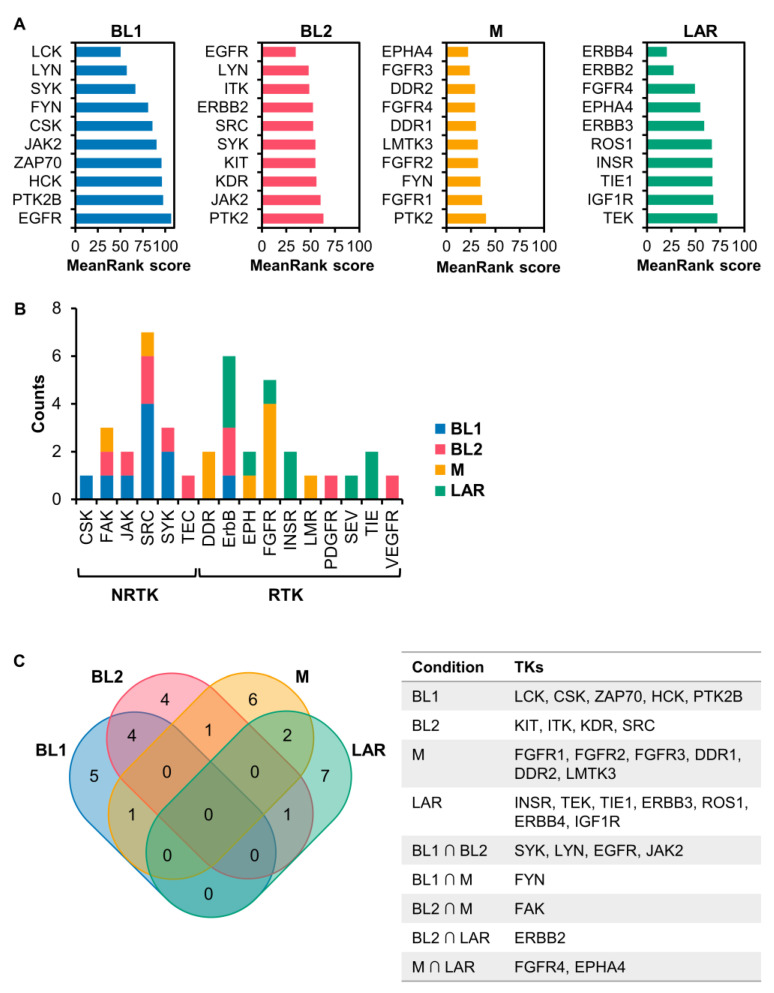
Kinase enrichment analysis. (**A**) Top ten predicted TKs from KEA3 based on MeanRank score. (**B**) Distribution of non-receptor (NRTKs) and receptor TKs (RTKs). TKs from (**A**) were grouped into subclass of the TK family. (**C**) Venn diagram of the top ten predicted TKs of each TNBC subtype. The information on TKs of TNBC subtypes was illustrated in the table.

To further investigate the relation of TKs on different subtypes of TNBC, only the top 10 TKs were investigated (Figure 3A,B). We found that the majority of TKs in BL1 were NRTKs with only one RTK (i.e., EGFR), while all TKs in LAR were RTKs. On the contrary, both NRTKs and RTKs can be found in BL2 and M (Figure 3B). These results indicated that DE genes encoding kinase proteins in BL2, M, and LAR were mainly involved in the signal transduction of the RTKs, but opposite to BL1. Considering the classes of TKs for all TNBC subtypes, only three were dominant, including the SRC family (i.e., FYN, HCK, LCK, LYN, and SRC), the ErbB family (i.e., EGFR or ERBB1, ERBB2, ERBB3, and ERBB4), and the FGFR family (i.e., FGFR1, FGFR2, FGFR3, and FGFR4), with different distribution in each TNBC subtype (Figure 3B). 

The most enriched kinases in each TNBC subtype were relatively unique with a small number of TKs overlapping between any two subtypes (Figure 3C). In BL1, NRTKs such as LCK, CSK, ZAP70, HCK, and PTK2B (PYK2), which are mainly involved in the regulation of the immune system, were enriched. In BL2, several enriched TKs were similar to those in BL1, including SYK, LYN, JAK2, and EGFR, which were reported to be associated with the aggressiveness of TNBC [25,38,39,40]. EGFR was found to be associated with BL2 more than BL1 based on the MeanRank score. The candidate signature TKs for BL2 were ITK, SRC, KIT, and KDR (VEGFR2). For M and LAR, unique TKs generally act as cell–surface receptors with different functions. Three major types of enriched RTKs in M were the FGFR family (i.e., FGFR1, FGFR2, and FGFR3), the DDR family (i.e., DDR1 and DDR2), and Lemur tyrosine kinase-3 (LMTK3). In LAR, diverse RTKs were identified, such as the ErbB family (i.e., ERBB3 and ERBB4), the insulin/insulin-like receptor family (i.e., INSR and IGF1R), the TIE receptor family (i.e., TIE1 and TEK (TIE2)), and ROS proto-oncogene 1 (ROS1). 

Comparing the results of predicted upstream kinases and subtype-specific TK genes of the TNBC subtype, we found that 10%, 40%, 60%, and 50% of TKs inferred by KEA3 were identity to the subtype-specific TK genes of the BL1, BL2, M, and LAR subtype, respectively. These overlapping TKs could also be used as the subtype-specific signature TKs of each TNBC subtype.

### 3.4. KEGG and GO Analysis of DE Genes

**Figure 4:**
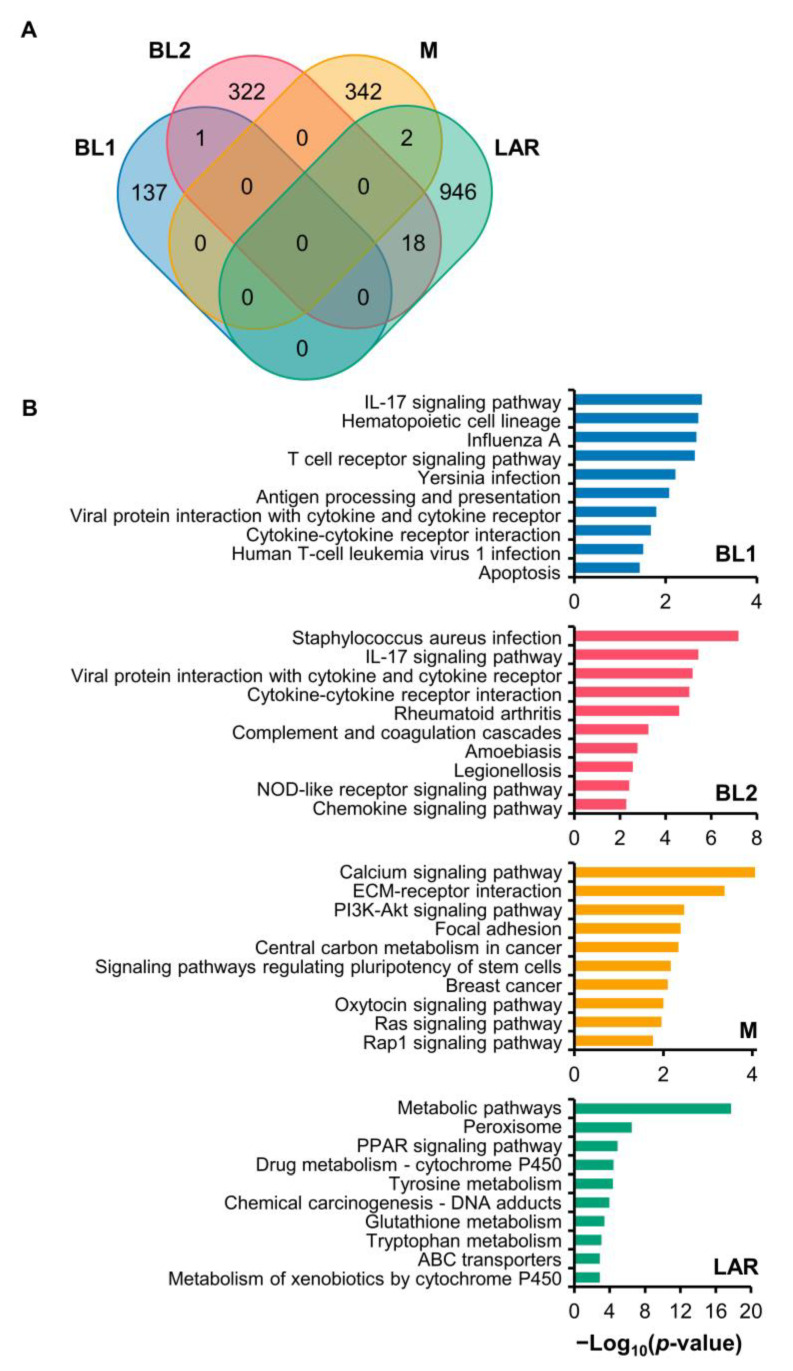
Gene expression and KEGG pathway enrichment analysis of four TNBC subtypes. (**A**) Venn diagram of the DE genes in the four subgroups of TNBC. Upregulated DE genes of the individual TNBC subtypes included BL1 (137 genes), BL2 (322 genes), M (342 genes), and LAR (946 genes) with minimal overlapping between subtypes. (**B**) KEGG pathway analysis enriched in upregulated genes of specific subtypes. The top ten enriched pathways were displayed with the minus of Log_10_ (*p*-value).

To investigate the unique biological functions of DE genes, functional enrichment analyses were performed using KEGG and GO analysis on unique and upregulated DE genes (Figure 4A). Only those with Fisher’s exact *p*-values < 0.05 were selected for further analysis. The top 10 pathways for KEGG and GO analyses were displayed in Figure 4B and Figure S3. 

KEGG pathway analysis of DE genes revealed differences between TNBC subtypes (Figure 4B). The basal-like subtype of TNBC, BL1 and BL2, had enrichment of genes mainly associated with the immune system and infectious disease with a few overlapping pathways, such as IL-17 signaling pathway, viral protein interaction with cytokine and cytokine receptor, and cytokine–cytokine receptor interaction. However, the pathway in BL1 was also related to cell growth and death, whereas that in BL2 was involved in the inflammatory immune response. In M, enriched signaling pathways were related to intracellular processes, and cellular functions, through several pathways, such as calcium, PI3K-Akt, oxytocin, Ras, and Rap1 signaling pathways, and the extracellular processes, such as focal adhesion and ECM-receptor interaction. In LAR, the DE genes were mostly associated with metabolism pathways, such as peroxisome, drug metabolism, tyrosine metabolism, glutathione metabolism, tryptophan metabolism, and metabolism of xenobiotics. Besides, the DE genes were in response to external cues, such as drugs or chemicals, through the PPAR signaling pathway, ABC transporters, and chemical carcinogenesis. Interestingly, even though the DE genes were enriched in diverse pathways, only the M subtype was found to be related to the breast cancer pathway.

GO analysis, including biological process (BP), cellular component (CC) and molecular function (MF) analysis, revealed distinct characteristics of each TNBC subtype (Figure S3). BP analysis showed that BL1 TNBC cancer exhibited a strong association with the immune system process and immune response to stimulus. Besides the immune system process, BL2 was found to be associated with the cell development process, including epidermis development, keratinization, and keratinocyte differentiation. The M subtype was linked with cell development, communication, and response to stimulus, while the LAR subtype was mainly involved in metabolic processes, such as drug metabolism. For CC analysis, the upregulated DE genes in BL1, BL2, and M were mainly enriched in the membrane, followed by the extracellular region. In contrast, the cytoplasm part was the dominant subcategory for LAR. Moreover, the changes in MF of BL1 were significantly enriched in serine hydrolase activity and carbohydrate binding. In BL2, functions related to signaling receptor activity, chemokine receptor binding, and signaling receptor binding were detected. In contrast, the dominant functions in M were protein TK activity, protein binding, and structural molecule activity. In LAR, on the other hand, the changes in MF were mainly associated with catalytic activity and ion binding.

### 3.5. Retrospective Validation of Signature TK Genes

To validate the potential signature TKs for each TNBC subtype, we compared our findings to the RNA expression of TKs in other TNBC patient and cell line datasets. Three datasets are from TNBC patients, including CPTAC (27 patients), MET500 (39 patients), and METABRIC (325 patients). Another dataset is from CCLE, including 20 TNBC cell lines. These datasets were then subcategorized into BL1, BL2, M, and LAR, according to previous reports [3,4,41]. Two assessment methods were used to validate our discovered subtype-specific TK genes in retrospect. 

**Figure 5:**
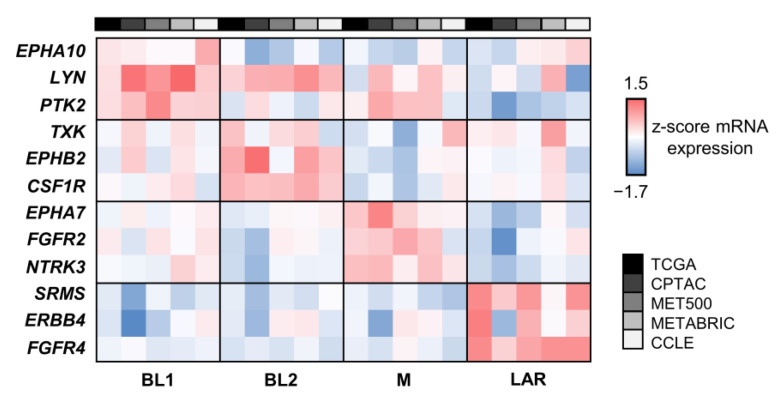
The comparison of the mRNA expression z-score of signature TK genes of each TNBC subtype. The heatmap displayed the mRNA expression z-score of the top three TKs that were unique in each TNBC subtype comparing among four TNBC datasets including TCGA, CPTAC, MET500, METABRIC, and CCLE. Each row represented DE TK genes, and each column represented the individual TNBC subtypes from different datasets. The color bar from blue to red showed the level of the mRNA expression z-score ascendingly.

The mRNA expression z-score of the top three subtype-specific signature TK genes of each TNBC subtype was used to compare the changes in the expression level of selected kinases within the same dataset, as well as to compare the trend across datasets (Figure 5). We found that the changes in expression of TK genes in each TNBC subtype were overall consistent to all datasets. TK genes with slightly positive FC in BL1, including *EPHA10*, *LYN*, and *PTK2,* could possibly be involved in this subtype since their RNA expression was the highest in BL1. Furthermore, the z-score of the top three kinases, including *TXK*, *EPHB2*, and *CSF1R* for BL2, *NTRK3*, *EPHA7*, and *FGFR2* for M, and *ERBB4*, *FGFR4*, and *SRMS* for LAR, were consistently the highest value in BL2, M, and LAR, respectively. We found that the RNA-expression of TKs from TCGA, CPTAC, MET500, METABRIC, and CCLE were mostly consistent, except for certain kinases, including *EPHA10*, *LYN*, *TXK*, and *FGFR2,* of which the z-score from METABRIC and CCLE did not agree well with the rest. The differences could be possibly due to the heterogenicity of TNBC, as well as experimental methods, such as TNBC microarray from METABRIC or small sample space of TNBC cell lines. Interestingly, signature TK genes in the LAR subtype showed distinguished RNA expression than other subtypes, indicating the promising unique signature of this specific subtype.

**Figure 6:**
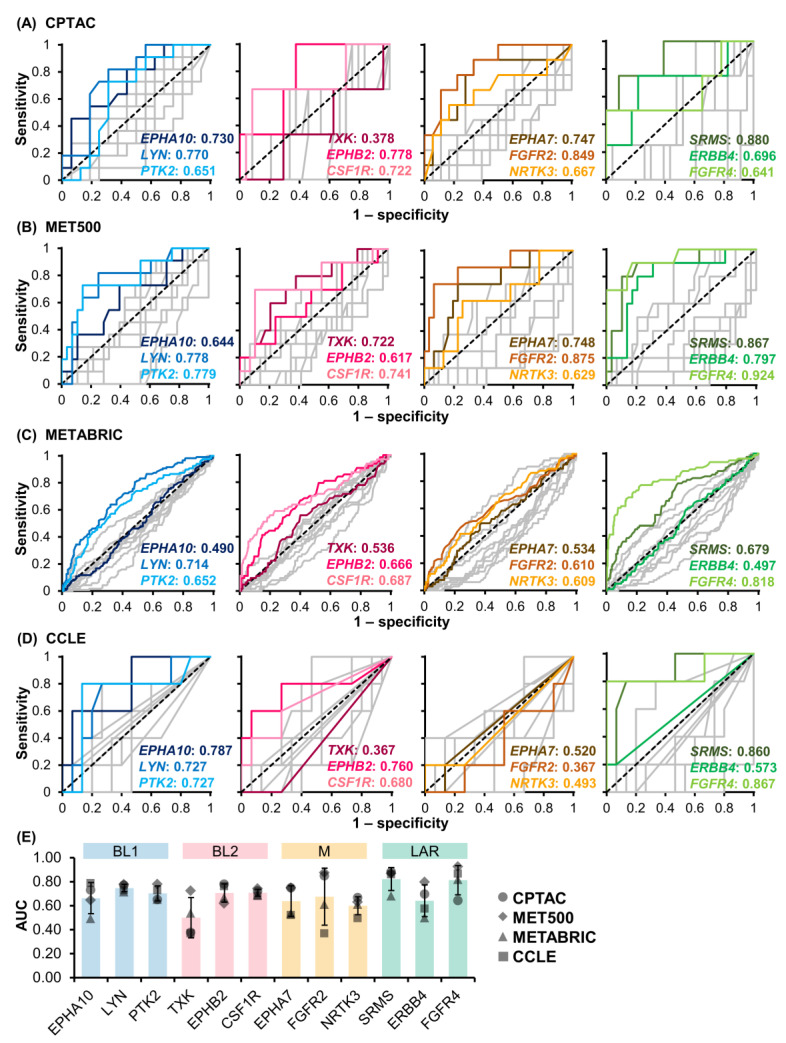
ROC analysis of signature TK genes in four datasets, including (**A**) CPTAC, (**B**) MET500, (**C**) METABRIC, and (**D**) CCLE. The blue, pink, orange, and green displayed the candidate signature TK genes in the BL1, BL2, M, and LAR subtypes, respectively, while grey lines illustrated the remaining TK genes. The area under the ROC curve (AUC) of each TK gene was shown in the lower right corner (TK gene:AUC). (**E**) The average AUC across the four datasets was summarized (mean ± standard deviation) with color and symbol according to the subtypes and datasets.

In addition, the sensitivity and specificity of our TNBC subtype-specific signature TK genes were evaluated using ROC analysis (Figure 6A–D). The AUC of the ROC curve was used to assess the performance of these TKs as a biomarker for identifying a specific TNBC subtype (Figure 6E). In BL1, the average AUC values of *EPHA10*, *LYN*, and *PTK2* were 0.633 ± 0.129, 0.747 ± 0.031, and 0.702 ± 0.063, respectively, where the individual AUC values of *LYN* and *PTK2* were similar across all four datasets. In BL2, the average AUC values of *TXK*, *EPHB2*, and *CSF1R* were 0.500 ± 0.167, 0.705 ± 0.076, and 0.708 ± 0.029, respectively. The individual AUC values of *EPHB2* and *CSF1R* were consistent across all datasets, while *TXK* performed poorly in CCLE, CPTAC, and METABRIC datasets. In M, the average AUC values of *EPHA7*, *FGFR2*, and *NRTK3* were 0.637 ± 0.128, 0.675 ± 0.237, and 0.599 ± 0.075, respectively. These M subtype-specific TK genes performed better in CPTAC and MET500 compared to METABRIC and CCLE datasets. Particularly, the AUC of *FGFR2* from CPTAC and MET500 datasets were 0.849 and 0.875, respectively. This indicated that *FGFR2* could be a potential biomarker for the M subtype. TK genes in LAR had the highest AUC values, especially *SRMS* and *FGRF4,* with average AUC values of 0.822 ± 0.095 and 0.813 ± 0.122, respectively, whereas *ERBB4* had a lower AUC value of 0.641 ± 0.135 due to inconsistent performance in CCLE and METABRIC datasets. In summary, eight out of 12 candidate subtype-specific TK genes could be potential biomarkers to classify TNBC subtypes, including *LYN* and *PTK2* for BL1, *CSF1R* for BL2, *EPHA7* and *FGFR2* for M, and *SRMS*, *ERBB4,* and *FGFR4* for LAR (average AUC > 0.700 from RNA-seq data of TNBC patients (CPTAC and MET500)). Among these TK genes, *FGFR2* for M and *SRMS* for LAR achieved the highest average AUC at 0.862 and 0.874, respectively.

## 4. Discussion

Tyrosine kinases are commonly involved in cancers and serve as important targets for anti-cancer therapies, as well as predictive markers for therapeutic response [9]. Because TNBC is currently treated as a single disease, identification of a TNBC subtype-specific signature TK gene could greatly improve the efficacy of the targeted treatment. Here, we focused on the expression of TKs in each TNBC subtype, including BL1, BL2, M, and LAR, to identify signature TKs that could be used as a prognostic marker and targeted therapy. We explored the TNBC TCGA RNA-seq dataset and discovered that each TNBC subtype expressed distinct TKs. Comparing our findings with other datasets of TNBC patients and cell lines, we found that the expression of these identified TKs among the TNBC subtypes had a similar trend across datasets. Moreover, the majority of these TKs exhibited good performance in classifying the TNBC subtypes as a result of the ROC analysis (AUC > 0.700). Thus, our findings can be used to create the TK mapping of TNBC subtypes, which will be important for both diagnosis and therapeutics. To investigate the subtype-specific TKs, two aspects of TK genes were considered, including the signature TK genes identified from upregulated differentially expressed TK genes and the upstream TKs predicted from the kinase enrichment analysis. 

For the DE TK genes, we found that the expression of these genes was unique in each subtype; therefore, they could be used as the predictive marker to distinguish the TNBC subtype. Although several evidences supported the high expression of our identified TKs in TNBC, and some of them have been under the clinical study [42], the signature TKs expressed in each TNBC subtype have not been reported. This information could be important for identifying a proper cancer therapy because each subtype responds to the drug differently. Overexpression of *EPHA10*, *LYN*, and *PTK2* (BL1 subtype-specific TK genes) is linked to the severity of breast cancer and can be used as the biomarker for TNBC [38,43,44,45]. LYN has been used as a potential drug target for TNBC reported by several studies [39,46]. Interestingly, LYN and PTK2 were found in the majority of TNBC cell lines classified as the BL1 subtype, which is consistent with our findings [38,47]. However, the signature TK genes in BL2 were still ambiguous. Only *CSF1R* and *EPHB2* (BL2 subtype-specific TK genes) were reported as having similar expression in different types of breast cancer, including TNBC [48,49]. Even though our study revealed that *TXK* has the highest FC of RNA-expression in BL2 among TNBC subtypes, the ROC analysis showed the opposite. Thus, the activity of TXK on breast cancer requires further investigation [50]. 

In addition, overexpression of *NTRK3*, *EPHA7*, and *FGFR2* (M subtype-specific TK genes) has been linked to a worse prognosis in breast cancer and could promote TNBC formation [51,52,53,54]. In the case of *ERBB4*, *FGRF4*, and *SRMS* (LAR subtype-specific TK genes), overexpression of ERBB4 and FGRF4 was reported to be associated with poor prognosis in TNBC [55,56]. Apart from ERBB4, another RTK in the ErbB family, ERBB2 (HER2), was upregulated in the LAR subtype, which is expected to be HER2-negative. This finding was consistent with the previous report that the occurrence of this breast cancer oncogene, ERBB2, is accompanied by overexpression of ERBB4 [57]. Moreover, the expression of ERBB4 and FGFR4 was detected in the LAR subtype of TNBC, which agrees well with our findings [58,59]. On the other hand, the non-receptor SRMS has not been reported on TNBC, but there is evidence supporting that it may serve as a biomarker for metastatic breast cancer [60]. Overall, some of our identified signature TK genes of each TNBC subtype have been reported by several studies as the potential biomarker for TNBC, but with limited information on the relationship to the TNBC subtype. Thus, more investigation of these TKs on an individual subtype of TNBC patients is needed.

Upregulated DE genes in each TNBC subtype were also used to predict the upstream kinases responsible for the observed differential phosphorylation. Out of more than 500 protein kinases, we found that TKs were dominant in the top-ranked predicted kinases of the TNBC subtype. This suggests that TKs could play an important role in the phosphorylation activities that contribute to signal transduction in TNBC. The basal-like subtype of TNBC showed the similarity to the enriched TKs. About half of them in BL1 and BL2 were overlapping, such as LYN, JAK2, SYK, and EGFR, which were reported to be associated with the aggressiveness of breast cancer, particularly in TNBC [25,38,39,40]. Interestingly, EGFR which is typically used as the marker for TNBC was significantly upregulated in BL2. Our results agreed well with previous studies that BL2 is highly involved in growth factor signaling pathways such as EGFR, while BL1 is involved in several cell activities [58]. In BL1, the enriched kinases, such as LCK and PTK2B, were associated with tumorigenesis, as well as invasion of breast cancer [61,62,63], whereas ZAP70 was reported to be related to drug resistance in TNBC [64]. In BL2, the predicted SRC and KIT have been recognized as proto-oncogenes and could be exploited as the targetable molecule for TNBC [65,66,67]. KDR (or VEGFR2) and PTK2 (or FAK) were observed in TNBC patients and could promote angiogenesis in TNBC cells [68]. Furthermore, the inhibition of ITK by Ibrutinib was reported to inhibit tumor development and metastasis in breast cancer [69]. 

For M and LAR, all unique TKs generally acted as cell–surface receptors with different functions. Three major types of enriched RTKs in M were FGFR, DDR, and LMTK3. The amplification of FGFR1, FGFR2, and FGFR3 was found in TNBC with variations associated with poor prognosis and overall survival [70,71]. DDR1 was revealed to control TNBC growth, while DDR2 overexpression could be a potential target for TNBC [72]. LMTK3 expression levels were implicated in cancer cell invasion, endocrine resistance, poor prognosis, and overall tumor progression in different types of malignancies [73], and its high expression has been observed in TNBC [74]. In addition, we found that other kinases from the ErbB family, such as ERBB3 and ERBB4, were uniquely expressed in the LAR subtype [57]. Particularly, ERBB4 expression could serve as a possible prognostic factor in advanced-stage TNBC [56]. Other TKs, such as TIE1 and TEK (or TIE2), were enriched in TNBC [4], and a proto-oncogene ROS1 was a regulator of cellular signal transduction pathway that mediates cell proliferation and migration and cell-to-cell communication. Again, although several studies have reported the association of these TKs to TNBC, no subtype-specific TKs of each TNBC have been revealed.

Despite about 40% correlation between expression levels of mRNA and protein, genes with differentially expressed mRNA have significantly higher correlations between mRNA and protein than those with non-differentially expressed mRNA [75,76]. Our findings revealed the correlation between the subtype-specific TK genes and the predicted upstream TKs. However, those kinases in BL1 showed a contradiction due to the low expression of BL1 subtype-specific TK genes in comparison to the rest of TNBC subtypes. In addition, our result showed the relationship between signature TKs and the biological functions in each TNBC subtype. We discovered that the identified subtype-specific kinases, predicted upstream kinases, and the outcome of the functional enrichment analysis are mainly coherent. The change in RNA expression of those TKs relatively agreed well across all validation datasets from both patients and cell lines. Furthermore, ROC analysis on RNA-seq data from TNBC patients showed excellent performance of our identified signature TKs in classifying TNBC subtypes, especially *FGFR2* for M and *SRMS* for LAR, with the highest average AUC > 0.860. However, the results from CCLE were particularly inconsistent. This is likely due to the difference in the microtumor environment surrounding TNBC cells between the cell line and the tumor cells within the patient’s body. Besides the heterogenicity of TNBC patients, the differences in experimental methodologies, such as RNA-seq (TCGA, CPTAC, MET500) and microarray (METABRIC), cause inconsistency in gene expression where we found that the results from TCGA, CPTAC, MET500 datasets were mainly consistent. Although TKs appear as potential candidates for personalized medicine, the plasticity and redundancy of the kinome present key challenges for drug development. Targeting this TK may result in the upregulation and system-wide changes in multiple TK expressions and activities. More investigation is still required to better verify these identified TKs prior clinical practice.

## 5. Conclusions

Analyzing transcriptomic TK profiles of TNBC from TCGA datasets and validating the findings with the other TNBC patient datasets reveals potential signature TKs of TNBC subtypes, which are uniquely and highly expressed, for future investigation. These subtype-specific TKs could serve as novel targets for clinical exploration and therapeutic application. Moreover, these identified TKs could be adapted as an additional staining procedure to distinguish the TNBC subtype.

## Data Availability

TCGA RNA-seq gene expression data was available through https://xenabrowser.net/ (accessed on 17 July 2022) (cohort: TCGA Pan-Cancer (PANCAN); dataset ID: tcga_RSEM_gene_tpm). CPTAC mRNA-seq z-score was available through https://www.cbioportal.org/datasets (accessed on 15 December 2022) (Breast Cancer (Proteogenomic landscape of breast cancer (CPTAC, Cell 2020) [33]). METABRIC mRNA microarray z-score was available through https://www.cbioportal.org/datasets (accessed on 25 September 2022) (Breast Cancer (METABRIC, Nature 2012 & Nat Commun 2016) [35,77,78]). MET500 RNA-seq z-score was available through https://xenabrowser.net/ (accessed on 25 September 2022) (cohort: MET500 (expression centric) [34]; dataset ID: MET500/geneExpression/M.mx.log2.zscore.txt). CCLE RNA-seq gene expression data was available through https://depmap.org/portal/download/ (accessed on 25 September 2022) (DepMap Public 22Q2: CCLE_expression.csv).

## Supplementary Materials

The following supporting information can be downloaded at: https://www.mdpi.com/article/10.3390/cancers15020403/s1, Figure S1. Relationship of multiple components of principle component analysis of TNBC subtype; Figure S2. The human kinome mapping of upstream TKs inferred by KEA3; Figure S3. GO enrichment analysis of subtype-specific DE genes.
