# Supplementary material for: Transcriptomic Analysis of Subtype-Specific Tyrosine Kinases as Triple Negative Breast Cancer Biomarkers
Source: Cancers (Basel). 2023 Jan 7;15(2):403. doi: 10.3390/cancers15020403 (PMC9856281; doi:10.3390/cancers15020403)
Supplement: Supplementary file 1 [file cancers-15-00403-s001.zip › cancers-2147013-supplementary.pdf]

Supplementary Material

Transcriptomic Analysis of Subtype-specific Tyrosine Kinases as Triple Negative Breast Cancer Biomarkers

Praopim Limsakul [1,2], Pongsakorn Choochuen [3], Gorn Charupanit [4,5] and Krit Charupanit [3,*]

1 Division of Physical Science, Faculty of Science, Prince of Songkla University, Songkhla, 90110, Thailand
2 Center of Excellence for Trace Analysis and Biosensor (TAB-CoE), Prince of Songkla University, Songkhla, 90110, Thailand
3 Department of Biomedical Sciences and Biomedical Engineering, Faculty of Medicine, Prince of Songkla University, Songkhla, 90110, Thailand
4 Department of Pathology, Faculty of Medicine, Chulalongkorn University, Bangkok, 10330, Thailand
5 Department of Pathology, Pattani Hospital, Pattani, 94000, Thailand
* Correspondence: krit.ch@psu.ac.th

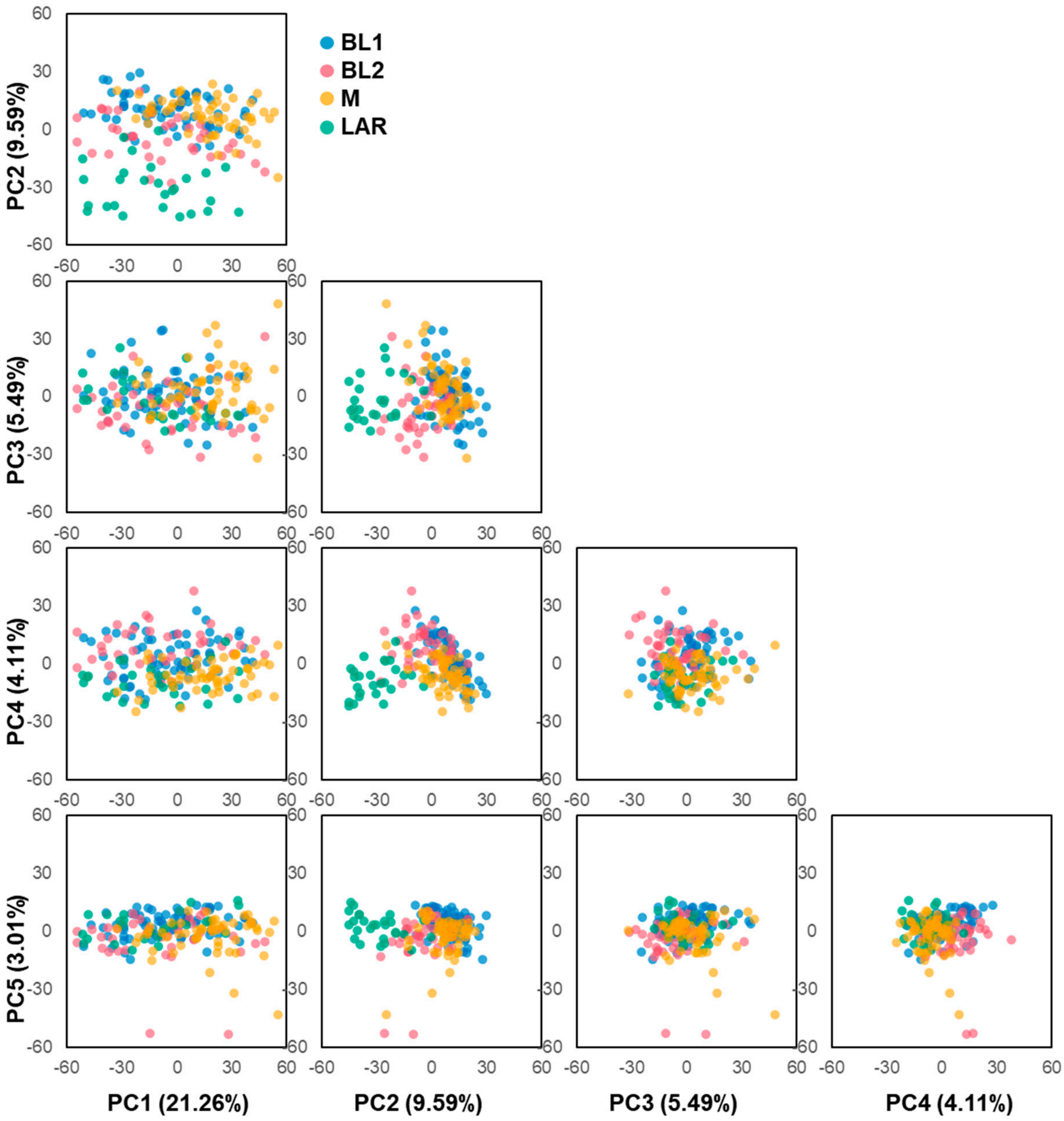

Figure S1. Relationship of multiple components of principle component analysis of TNBC subtype.

Principal component analysis of mRNAs expression in each subtype including BL1 (blue), BL2 (pink), M (orange), and LAR (green). Each dot represented one TNBC cohort.

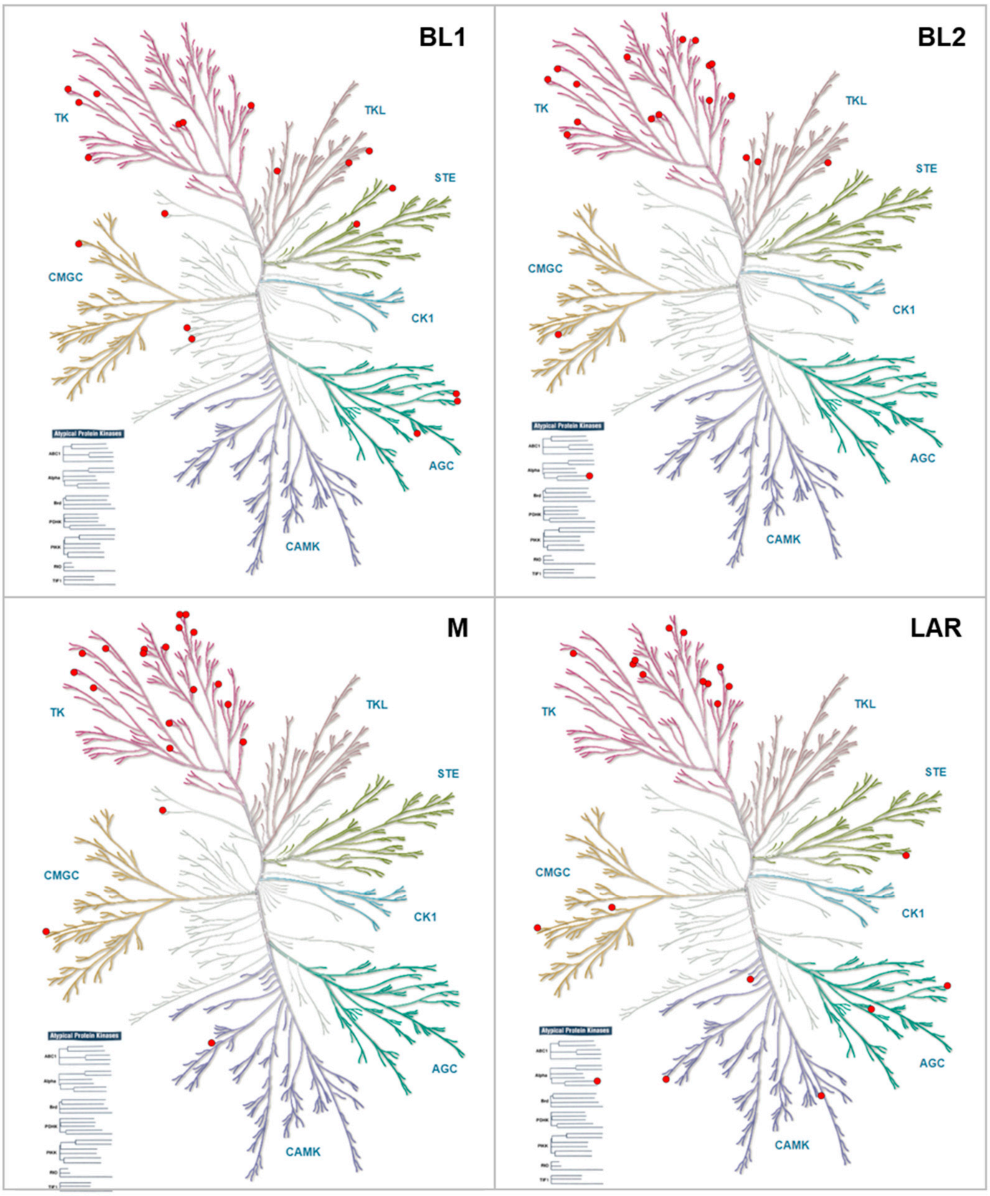

Figure S2. The human kinome mapping of upstream TKs inferred by KEA3.

The kinome trees illustrated the top 20 enriched TKs in each TNBC subtype predicted by KEA3. The kinases were separated into eight typical groups (AGC, CAMK, CK1, CMGC, STE, TK, TKL, Other) and 13 atypical families among the human kinome. The kinome tree illustration was adapted with permission from Cell Signaling Technology, Inc.

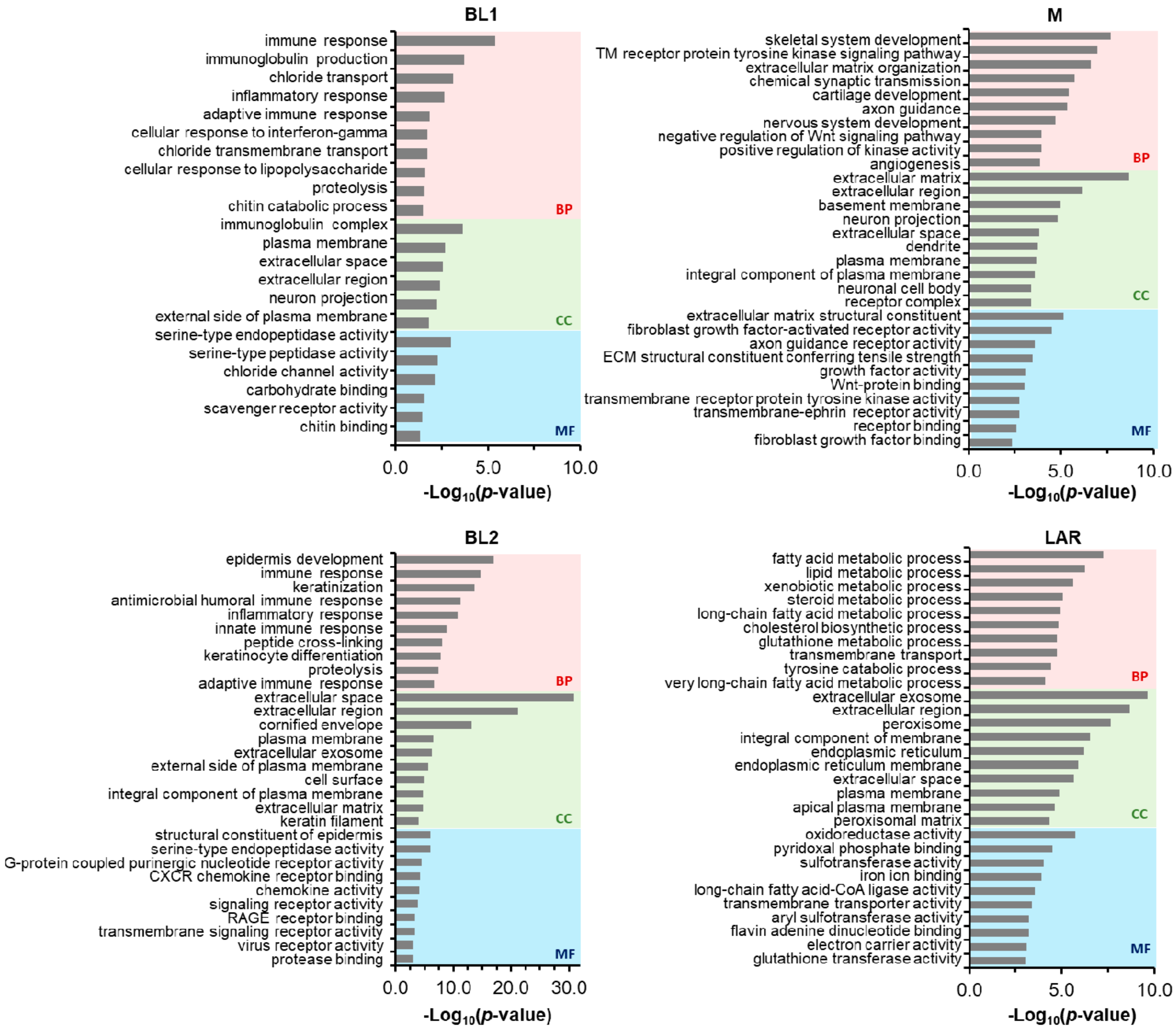

Figure S3. GO enrichment analysis of subtype-specific DE genes.
